# Effects of Early Physical Therapy and Follow-Up in Acute Severe Coronavirus Disease 2019 Pneumonia: A Retrospective Observational Study

**DOI:** 10.3389/fmed.2022.866055

**Published:** 2022-04-11

**Authors:** Jaume Bordas-Martínez, Ana Luzardo-González, Alejandro Arencibia, Franco Tormo, Lluís Matéu, Vanesa Vicens-Zygmunt, Guadalupe Bermudo, Salud Santos, María Molina-Molina, Rosa Planas, Guillermo Suarez-Cuartín

**Affiliations:** ^1^Respiratory Department, Bellvitge University Hospital, IDIBELL, Hospitalet de Llobregat, University of Barcelona, Barcelona, Spain; ^2^Rehabilitation Department, Bellvitge University Hospital, IDIBELL, Hospitalet de Llobregat, University of Barcelona, Barcelona, Spain

**Keywords:** early physical therapy, rehabilitation, pulmonary rehabilitation, COVID-19, SARS-CoV-2

## Abstract

**Background:**

Rehabilitation in subjects with severe coronavirus disease 2019 (COVID-19) pneumonia has been widely recommended. However, data regarding the starting time of rehabilitation, subjects and healthcare workers’ safety, as well as rehabilitation program features are limited. We aimed to assess the safety and characterize the effect of early and non-early physiotherapy on severe COVID-19 pneumonia subjects.

**Methods:**

A retrospective cohort study, including a consecutive sample of surviving subjects admitted to an acute care hospital due to severe COVID-19 pneumonia from March 13th to May 15th of 2020, is made. Subjects were separated into three groups: non-physical therapy, early physiotherapy (onset <7 days of admission), and non-early physiotherapy. Subject and therapist safety and length of hospital stay were the main evaluated outcomes.

**Results:**

A total of 159 subjects were included (72% men; median age 62 years). Rehabilitation was performed on 108 subjects (32 early and 76 non-early physiotherapies). The length of hospital stay was 19 [interquartile range (IQR) 36.25] and 34 days (IQR 27.25) (*p* = 0.001) for early and non-early physiotherapy groups, respectively. No physiotherapist was infected and no subject adverse effect was identified. Multivariate analysis of subjects receiving physiotherapy during admission identified obesity [odds ratio (OR) 3.21; *p*-value 0.028], invasive mechanical ventilation (OR 6.25; *p*-value <0.001), and non-early physiotherapy (OR 3.54; *p*-value 0.017) as independent factors associated with a higher risk of prolonged hospital stay. Survivors’ follow-up after hospital discharge at 8 weeks was completed by 54% of subjects.

**Conclusion:**

Rehabilitation in acute severe COVID-19 pneumonia is safe for subjects and healthcare workers and could reduce the length of hospitalization stay, especially in those that may start early.

## Introduction

Coronavirus disease 2019 (COVID-19) is produced by the infection of a virus of the coronavirus family [severe acute respiratory syndrome coronavirus 2 (SARS-CoV-2)] identified in December 2019 in China (1). Patients with COVID-19 could develop severe pneumonia in 20% of the cases (2, 3), which is defined by radiographic pneumonia plus respiratory rate >30 breaths⋅min^–1^ or severe respiratory distress or oxygen saturation measured by pulse oximetry (*S*_*p*_O_2_) ≤93% on room air at rest (1, 4). Furthermore, 5% of patients with COVID-19 pneumonia may develop multiple organ dysfunction requiring intensive care unit (ICU) admission (2, 3, 5, 6) and life support, including mechanical ventilation, for prolonged periods (2). The most frequent ICU complications related to COVID-19 are acute respiratory distress syndrome (ARDS), in up to 29% of patients (2), and intensive care unit (ICU)-acquired weakness (ICUAW) (7).

Intensive care unit-acquired weakness is a critical illness characterized by polyneuropathy, myopathy, and neuromyopathy that may develop in approximately 40% of patients requiring mechanical ventilation (8–10). In previous coronavirus epidemics, severe acute respiratory syndrome, and middle east respiratory syndrome, around 50% of subjects presented a significant decrease of diffusion capacity, limitation of exercise capacity, and decreased quality of life after several months of hospital discharge (11–14). The first available data related to post-COVID-19 consequences show abnormal pulmonary function and muscle weakness, related to subject functional limitation in their everyday lives (15, 16), especially in elderly patients (17). In this regard, rehabilitation has shown lung function improvement and quality of life recovering in ICU survivors from other diseases (18) and ARDS fatigue (19).

Early rehabilitation has been associated with a reduction of the likelihood of developing ICUAW (20). Initially, rehabilitation in patients with COVID-19 (1, 4) was not performed due to subject and medical staff safety concerns, as there is a possibility of generating airborne aerosols of SARS-CoV-2 particles (21, 22). Undoubtedly, the healthcare work overload and the lack of personal protection equipment at the beginning of the pandemic also played a role (5). However, following the recommendations of main medical societies (23–26) and experts (27–30), early rehabilitation was initiated in stable subjects. Previous data on subacute rehabilitation in COVID-19 have reported promising clinical benefits with no adverse effects reported on either subjects or medical staff (7, 31–34). Nevertheless, studies exploring the early initiation of physical therapy are lacking. Therefore, this study evaluated the impact of rehabilitation on severe COVID-19 pneumonia survivors and compared the potential benefits depending on the time of therapy initiation.

## Materials and Methods

### Study Design

This is a single-center retrospective cohort observational study with prospective follow-up at hospital discharge. The STROBE guidelines (35) for observational studies were followed. Inclusion criteria were: (1) survival of severe COVID-19 pneumonia; (2) requirement of high oxygen support required [inspired oxygen fraction (FiO_2_) >0.5] with high-flow nasal cannula (HFNC), and/or either invasive (IOT-MV) or non-invasive (NIV) mechanical ventilation (MV). All the consecutive patients admitted to the ICU and/or the intermediate respiratory care unit (IMCU) from March 13th until May 15th of 2020 were included. COVID-19 laboratory-diagnostic was performed using nasopharyngeal swab PCR.

According to rehabilitation performance and time of onset, subjects were retrospectively divided into two groups: non-physical therapy and physical therapy, which was further divided into early physiotherapy (defined by therapy start during the first 7 days after admission) and non-early physiotherapy groups. Subjects were followed-up by a pulmonologist with a telephone visit and chest X-ray at 30–60 days of discharge.

This study was approved by the Hospital Ethics Committee (reference code PR168/20). Informed consent of prospective follow-up after hospital discharge was verbally given and appropriately registered in the medical record.

### Rehabilitation Program

Rehabilitation was carried out in the ICU and IMCU. The time of therapy initiation was decided according to patient clinical stability (23–25, 36) and the availability of physiotherapists. Not all eligible patients could perform early rehabilitation due to a lack of resources.

The requirements for starting physical therapy were as follows: (1) subject is conscious, oriented, and collaborative; (2) hemodynamically stable without vasoactive support drugs; (3) respiratory rate ≤30 breaths/minute; (4) FiO_2_ requirement ≤0.6 for SpO_2_ ≥90%; and (5) positive end-expiratory pressure (PEEP) ≤10 cm H_2_O. The rehabilitation session would stop if there was one of the following events: (1) hemodynamic instability; (2) oxygen desaturation lower than 90%; (3) dyspnea higher than 4 points on the Borg scale; and (4) incoercible cough, nausea, or other limiting adverse effects.

Pulmonary and musculoskeletal rehabilitation was performed 5–7 days per week according to physiotherapist availability in ICU and IMCU. Each exercise was repeated between 1 and 10 times depending on patient tolerance with a moderate intensity (never exceeding 4 on dyspnea Borg scale or tachycardia greater than 130 beats per minute). The length of the sessions was 10–15 min. The rehabilitation program involved muscle strength and endurance, inspiratory/expiratory muscle training, guided ventilations, autogenous drainage, and positive expiratory pressure (PEP) exercises. Devices, such as PEP-bottle (37) or Threshold PEP (38), were used when required.

Further information on the rehabilitation program, safety measures, and exercise programs is available in Supplementary Material.

### Data Collection

Demographic collected variables were gender, age, and comorbidities, such as hypertension, diabetes mellitus, dyslipidemia, obesity, heart disease, lung disease, kidney disease, liver disease, and cancer history. Collected clinical data of severe pneumonia were survival time, admission and hospital discharge date, intensive and intermediate critical care requirements with ventilatory and oxygenation support, laboratory serum data [albumin, C-protein reactive (CRP), ferritin, lactate dehydrogenase (LDH), and D-dimer], and pharmacological treatment. The number of physiotherapy sessions, subject adverse events, and physiotherapist COVID-19 contagion were recorded as well. A patient adverse event was considered when the following situations were observed: cardiovascular or respiratory worsening, coughing attacks, or hemodynamic instability. Physical therapist adverse event was considered whether COVID-19 infection was detected or work-related injury was produced due to COVID-19 isolation in ICU or IMCU.

Follow-up was prospectively recorded between 30 and 60 days of hospital discharge including chest X-ray evolution, general status EuroQoL visual analog scale (EQ-VAS) (39), modified medical research council (mMRC) (40) dyspnea scale, and clinical data, such as dry and productive cough, chest pain, palpitations, and neuropathy symptoms.

### Statistical Analysis

Statistical analysis was performed using R (software version 3.6.2). Data in the descriptive analysis were presented as frequency (*n*) and percentage (%) for categorical variables, while as mean and SD or median and interquartile range (IQR) for continuous variables, depending on the normal or non-normal distribution of the variable, respectively. Comparative statistical tests were used for the chi-squared test or Fisher’s exact test was used when appropriated. A subgroup analysis was performed only in subjects that underwent physiotherapy during admission to assess the impact of early versus non-early physiotherapy on the length of hospital stay. Prolonged hospitalization was defined as more than 28 days of admission. We performed a multivariate analysis including clinically significant variables. The final model was chosen by Akaike Information Criterion in a stepwise algorithm. A value of *p* < 0.05 was considered statistically significant. The STROBE initiative recommendations were used (41).

## Results

All the 208 severe COVID-19 subjects who survived the acute phase and were admitted to the ICU or IMCU from March 13th until May 15th of 2020 were included. Of them, 49 who did not require HFNC or MV support were excluded. Of the 159 subjects analyzed, 72% were men and the median age was 61 years old. Hypertension (50%) was the most frequent comorbidity, followed by dyslipidemia (44%), obesity (29%), and diabetes mellitus (27%). Physical therapy was performed in 108 subjects (68%). These subjects were significantly older than those in the non-physiotherapy group (62 vs. 55 years old; *p*-value 0.01), and developed a more severe disease with longer ICU admission time, mechanical ventilation, and tracheostomy requirement, as well as worse laboratory profile (low minimum albumin, higher maximum CRP, LDH, and D-dimer blood levels). No physiotherapist was infected by COVID-19 and no adverse effect or worsening in subjects was detected in our study. The non-physical therapy group required significantly less time than the physical therapy group from admission to achieving the sitting position than the physical therapy group (10 vs. 19 days; *p* < 0.001) and to hospital discharge (14 vs. 31 days, *p* < 0.001). Subject characteristics are shown in Table 1.

**TABLE 1:** Subjects characteristics.

	All subjects (n 159)	Non-physiotherapy group (*n* = 51)	Physiotherapy group (*n* = 108)	*p*-value
Age, median (IQR)	61 (17)	55 (19)	62 (15)	*0.010* *
Male gender, n (%)	115 (72.33%)	35 (68.63%)	80 (74.07%)	0.598
Hypertension, n (%)	79 (49.69%)	20 (45.75%)	59 (54.63%)	0.101
Diabetes, n (%)	43 (27.04%)	10 (19.61%)	33 (30.56%)	0.208
Dyslipidemia, n (%)	70 (44.03%)	21 (41.18%)	49 (45.37%)	0.744
Obesity, n (%)	46 (28.93%)	11 (21.57%)	35 (32.41%)	0.223
Cardiopathy, n (%)	11 (6.92%)	1 (1.96%)	10 (9.26%)	0.175
Chronic respiratory disease, n (%)				
-Asthma	6 (3.77%)	2 (3.92%)	4 (3.70%)	0.849
-COPD	6 (3.77%)	3 (5.88%)	3 (2.78%)	
-Bronchiectasis	2 (1.26%)	0	2 (1.85%)	
-OSA	8 (5.03%)	2 (3.92%)	6 (5.56%)	
History of malignancies, n (%)	19 (11.95%)	7 (13.73%)	12 (11.11%)	0.832
Hepatopathy, n (%)	12 (7.55%)	5 (9.80%)	7 (6.48%)	0.675
Chronic renal disease, n (%)	8 (5.03%)	3 (5.88%)	5 (4.63%)	0.712
Immunosuppression, n (%)	2 (1.26%)	0	2 (1.85%)	0.829
Admission ward, n (%)				
-General ward	65 (40.88%)	27 (52.94%)	38 (35.19%)	*0.003* *
-IMCU	59 (37.11%)	21 (41.18%)	38 (35.19%)	
-ICU	35 (22.01%)	3 (5.88%)	32 (29.62%)	
High-flow oxygen, n (%)	130 (81.76%)	45 (88.24%)	85 (78.70%)	0.218
Non-invasive ventilation, n (%)	77 (48.43%)	15 (29.41%)	62 (57.41%)	*0.002* *
Invasive mechanical ventilation, n (%)	73 (45.91%)	7 (13.73%)	66 (61.11%)	<0.001*
Tracheostomy, n (%)	30 (18.99%)	0	30 (28.04%)	<0.001*
Lopinavir/ritonavir, n (%)	116 (72.96%)	41 (80.39%)	75 (69.44%)	0.208
Hydroxychloroquine, n (%)	157 (98.74%)	50 (98.04%)	107 (99.07%)	> 0.999
Interferon beta-1b, n (%)	55 (34.59%)	18 (35.29%)	37 (34.26%)	> 0.999
Tocilizumab, n (%)	74 (46.54%)	27 (52.94%)	47 (43.52%)	0.346
Systemic corticosteroids, n (%)				
-IV bolus	66 (41.51%)	25 (49.02%)	41 (37.96%)	*0.029* *
-IV bolus + oral descending regimen	43 (27.04%)	8 (15.69%)	35 (32.41%)	
-Oral descending regimen	6 (3.77%)	0	6 (5.56%)	
Remdesivir, n (%)	12 (7.55%)	0	12 (11.11%)	*0.031* *
Deep venous thrombosis, n (%)	7 (4.40%)	1 (1.96%)	6 (5.56%)	0.431
Pulmonary thrombosis, n (%)	12 (7.55%)	2 (3.92%)	10 (9.26%)	0.340
Pneumothorax, n (%)	4 (2.52%)	2 (3.92%)	2 (1.85%)	0.594
Subjects situation, n (%)				
-Home discharge	83 (52.20%)	32 (62.75%)	51 (47.22%)	0.135
-Socio-health center discharge	63 (39.62%)	17 (33.33%)	46 (42.59%)	
-Regional hospital transfer	13 (8.18%)	2 (3.92%)	11 (10.19%)	
Days from admission to sitting position, median (IQR)	14.50 (19)	10 (7)	19 (23.35)	<0.001*
Length of hospital stay in days, median (IQR)	22 (26)	14 (7)	31 (31)	<0.001*
Minimum albumin (g/L), mean (SD)	27.21 (5.08)	30.41 (4.18)	25.68 (4.77)	<0.001*
Albumin on discharge (g/L), median (SD)	35.75 (3.97)	35.94 (4.25)	35.66 (3.85)	0.694
Maximum ferritin (μg/L), median (IQR)	1794 (1635.75)	1623.80 (1267.25)	1930 (1892.5)	0.059
Maximum CRP (mg/L), mean (SD)	253.30 (131.92)	201.60 (109.95)	277.7 (134.82)	<0.001*
Maximum LDH (U/L), median (IQR)	497 (251)	434 (187)	537.5 (261.25)	<0.001*
Maximum D-dimer (μg/L), median (IQR)	2111 (4663.25)	1027 (2937)	2879 (4975.25)	*0.004* *

IQR, Interquartile range; SD, Standard deviation; COPD, Chronic obstructive pulmonary disease; ILD, Interstitial lung disease; OSA, Obstructive sleep apnea; IMCU, Intermediate respiratory care unit; ICU, Intensive care unit; IV, Intravenous; CRP, C-reactive protein; LDH, Lactate dehydrogenase. * Represents significant results with p-value ≤ 0.05.

Among 108 subjects who underwent physical therapy, 32 (30%) started on the first 7 days of admission (early) and 76 (70%) started after the first week of admission (non-early). Their characteristics are depicted in Table 2. Initial admission to the ICU was higher in the early physiotherapy group (56% early vs. 18% non-early; *p*-value <0.001), who had shorter time from admission until physical therapy started (4 vs. 13 days, respectively; *p* < 0.001) and sitting position (9.5 vs. 22 days; *p* < 0.001). The length of hospital stay was 19 and 34 days (IQR 36.25 vs. 27.25; *p* = 0.001) in early and non-early physiotherapy groups, respectively.

**TABLE 2:** Subjects characteristics according to physiotherapy category.

	Early physiotherapy (*n* = 32)	Non-early physiotherapy (*n* = 76)	*p*-value
Age, median (IQR)	59.50 (14.25)	62.50 (18)	0.009
Male gender, n (%)	23 (71.88%)	57 (75%)	0.922
Hypertension, n (%)	13 (40.63%)	46 (60.53%)	0.091
Diabetes, n (%)	8 (25%)	25 (32.90%)	0.559
Dyslipidemia, n (%)	12 (37.50%)	37 (48.68%)	0.393
Obesity, n (%)	14 (43.75%)	21 (27.63%)	0.159
Cardiopathy, n (%)	4 (12.50%)	6 (7.90%)	0.478
Chronic respiratory disease, n (%)			
-Asthma	1 (3.13%)	3 (3.95%)	0.890
-COPD	0	3 (3.95%)	
-Bronchiectasis	0	2 (2.63%)	
-OSA	2 (6.25%)	4 (5.26%)	
History of malignancies, n (%)	5 (15.63%)	7 (9.21%)	0.334
Hepatopathy, n (%)	3 (9.38%)	4 (5.26%)	0.421
Chronic renal disease, n (%)	1 (3.13%)	4 (5.26%)	> 0.999
Immunosuppression, n (%)	0	2 (2.63%)	0.885
Admission ward, n (%)			
-General ward	6 (18.75%)	32 (42.11%)	< 0.001*
-IMCU	8 (25%)	30 (39.47%)	
-ICU	18 (56.25%)	14 (18.42%)	
High-flow oxygen, n (%)	25 (78.13%)	60 (78.95%)	> 0.999
Non-invasive ventilation, n (%)	18 (56.25%)	44 (57.90%)	> 0.999
Invasive mechanical ventilation, n (%)	20 (62.50%)	46 (60.53%)	> 0.999
Tracheostomy, n (%)	8 (25%)	22 (29.33%)	0.824
Lopinavir/ritonavir, n (%)	20 (62.50%)	55 (72.37%)	0.431
Hydroxychloroquine, n (%)	32 (100%)	75 (98.68%)	> 0.999
Interferon beta-1b, n (%)	11 (34.38%)	26 (34.21%)	> 0.999
Tocilizumab, n (%)	9 (28.13%)	38 (50%)	0.059
Systemic corticosteroids, n (%)			
-IV bolus	13 (40.63%)	28 (36.84%)	0.448
-IV bolus + oral descending regimen	7 (21.88%)	28 (36.84%)	
-Oral descending regimen	2 (6.25%)	4 (5.26%)	
Remdesivir, n (%)	6 (18.75%)	6 (7.90%)	0.192
Deep venous thrombosis, n (%)	1 (3.13%)	5 (6.58%)	0.667
Pulmonary thrombosis, n (%)	1 (3.13%)	9 (11.84%)	0.276
Pneumothorax, n (%)	0	2 (2.63%)	> 0.999
Subjects situation, n (%)			
-Home discharge	15 (46.88%)	36 (47.37%)	0.683
-Socio-health center discharge	15 (46.88%)	31 (40.79%)	
-Regional hospital transfer	2 (6.25%)	9 (11.84%)	
Place of physiotherapy start, n (%)			
-ICU	23 (71.88%)	42 (55.26%)	0.161
-IMCU	9 (28.12%)	28 (36.84%)	
-General ward	0	6 (7.90%)	
Days from admission to physiotherapy, median (IQR)	4 (2.25)	13 (8.25)	< 0.001*
Number of physiotherapy sessions, median (IQR)	20.50 (51.75)	23 (45)	0.284
Respiratory secretions, n (%)	10 (31.25%)	30 (39.47%)	0.555
Days from admission to sitting position, median (IQR)	9.50 (24)	22 (20)	< 0.001*
Length of hospital stay in days, median (IQR)	19 (36.25)	34 (27.25)	0.001*
Albumin on admission (g/L), mean (SD)	32.09 (5.54)	32.80 (4.99)	0.179
Minimum albumin (g/L), mean (SD)	26.06 (5.62)	25.53 (4.41)	0.212
Albumin on discharge (g/L), median (SD)	34.94 (4.02)	35.96 (3.77)	0.953
Maximum ferritin (μg/L), median (IQR)	1863 (1318.75)	2100 (2166.25)	0.288
Maximum CRP (mg/L), mean (SD)	248.8 (126.09)	289.80 (137.31)	0.123
Maximum LDH (U/L), median (IQR)	452 (180)	599 (291.50)	< 0.001*
Maximum D-dimer (μg/L), median (IQR)	1259.50 (3380.75)	3283 (4655.25)	0.021*

IQR, Interquartile range; SD, Standard deviation; COPD, Chronic obstructive pulmonary disease; ILD, Interstitial lung disease; OSA, Obstructive sleep apnea; IMCU, Intermediate respiratory care unit; ICU, Intensive care unit; IV, Intravenous; CRP, C-reactive protein; LDH, Lactate dehydrogenase. * Represents significant results with p-value ≤ 0.05.

Multivariate analysis showed that factors independently associated with a higher risk of prolonged hospitalization were obesity [odds ratio (OR) 3.21; 95% CI 1.18–9.66], invasive mechanical ventilation (OR 6.25; 95% CI 2.37–18.33), and starting physiotherapy after the first week of admission (OR 3.54; 95% CI 1.29–10.44) (Table 3).

**TABLE 3:** The multivariate analysis of prolonged hospitalization (>28 days) of subjects receiving physiotherapy during admission.

	Odds ratio	95% confidence interval	*p*-value
Age ≥70 years	2.59	0.87–8.55	0.099
Obesity	3.21	1.18–9.66	*0.028* *
Chronic renal disease	10.56	1.30–227.57	0.051
Hepatopathy	0.15	0.01–1.11	0.105
Invasive mechanical ventilation	6.25	2.37–18.33	<0.001*
Non-early physiotherapy	3.54	1.29–10.44	*0.017* *

* Represents significant results with p-value ≤ 0.05.

No significant differences were observed at 60 days follow-up between early and non-early physical therapy groups (Table 4). Oxygen therapy and oral corticosteroid treatment could be reduced at follow-up. Both groups reported dyspnea mMRC 1, neuropathy symptoms, and cough as the most frequent complaints at follow-up.

**TABLE 4:** Subjects characteristics at 60 days from discharge follow-up.

	Early physiotherapy (*n* = 25)	Non-early physiotherapy (*n* = 61)	*p*-value
Age, median (IQR)	57 (101)	62 (17)	0.051
Male gender, n (%)	19 (76%)	46 (75.41%)	> 0.999
High-flow oxygen during admission, n (%)	19 (76%)	47 (77.05%)	> 0.999
Non-invasive ventilation during admission, n (%)	14 (56%)	37 (60.66%)	0.875
Invasive mechanical ventilation during admission, n (%)	13 (52%)	33 (54.10%)	> 0.999
Tracheostomy during admission, n (%)	4 (16%)	13 (21.31%)	0.768
Oxygen therapy at discharge, n (%)	5 (20%)	7 (11.48%)	0.319
Oxygen therapy at follow-up, n (%)	2 (8%)	3 (4.92%)	0.625
Oral prednisone at discharge, n (%)	12 (48%)	33 (54.10%)	0.782
Oral prednisone at follow-up, n (%)	7 (28%)	22 (36.07%)	0.640
Chest X-ray infiltrates at follow-up, n (%)	14 (63.64%)	28 (49.12%)	0.364
Euro QoL VAS, median (IQR)	7 (3)	7.50 (2)	0.972
Walking capacity, n (%)			
- Completely limited	1 (4%)	0	0.074
- Limited to the bedroom	0	0	
- Limited to own home	2 (8%)	1 (1.67%)	
- No limitations	22 (88%)	60 (98.333%)	
Dyspnea mMRC scale at follow-up, median (IQR)	1 (1)	1 (1)	0.857
Cough, n (%)	7 (28%)	10 (16.67%)	0.248
Sputum production, n (%)	2 (8%)	6 (10%)	> 0.999
Chest pain, n (%)	3 (12%)	6 (10%)	0.719
Palpitations, n (%)	1 (4%)	3 (5%)	> 0.999
Neuropathy, n (%)	8 (32%)	16 (27.12%)	0.850

*IQR, Interquartile range; mMRC, Modified Medical Research Committee.*

## Discussion

This retrospective study evaluates the features of 108 subjects that started physiotherapy after surviving severe COVID-19 pneumonia and compared the potential benefits depending on the delay in starting this therapy (early versus non-early). The initiation of physical therapy was limited by available resources and safety concerns for subjects and healthcare workers during the first weeks of the pandemic. First and foremost, physical therapy was safe without physiotherapist infection and no adverse effect or worsening in subjects detected. This is consistent with previous rehabilitation studies in critical care units (42), as well as in reported acute (7) and postacute (32, 34, 43–46) COVID-19 rehabilitation studies. Therefore, physical therapy appears to be safe for subjects and healthcare workers.

On the other hand, the benefit of early systemic mobilization in critically ill subjects remains unproven, awaiting longer randomized trials (47), although it is being recommended (17, 28). In this regard, available data on the time of rehabilitation initiation on patients with acute severe COVID-19 pneumonia are still limited (48), with an average of 14 ± 7 days to first mobilization (7). In our study, physiotherapy was initiated within a median of 4 (IQR 2.25) and 13 days (IQR 8.25) from admission in the early and non-early groups of our severe COVID-19 cohort, respectively.

Interestingly, the time from admission until sitting position and the length of hospitalization were significantly shorter (12.5 and 15 days, respectively) in the early physical therapy group. Mc William et al. (7) identified that obesity and older patients were associated with a delay in time to the first mobilization in patients with acute severe COVID-19. Furthermore, another survivor cohort study of severe COVID-19 pneumonia showed that ventilation requirement was associated with a longer hospital stay (43). Similarly, the analysis of prolonged hospitalization performed in our subjects that underwent physiotherapy during admission identified that obese subjects, those who required invasive mechanical ventilation, and those who did not initiate early physiotherapy had higher probabilities of prolonged hospital stay.

Recent pulmonary rehabilitation studies in the post-acute phase of COVID-19 pneumonia reported significant benefits in exercise performance, lung function, and reported quality of life (32, 34, 43–46). In our study, follow-up of subject outcomes did not show differences between early and non-early physical therapy groups in terms of dyspnea, walking capacity, reported quality of life, and persistent chest X-ray infiltrates. However, this study lacks a control group without physical therapy to better assess its benefits. Both early and non-early physical therapy groups showed a decrease in oxygen therapy and corticosteroids requirements at 2 months of follow-up. Neuropathy and cough were the most frequently reported symptoms in both groups. Other COVID-19 postdischarge cohorts (49, 50) reported less fatigue, breathlessness, and better-reported quality of life in those subjects without ICU admission.

### Study Limitations

This study has several limitations and the conclusions have to be taken with caution. First, this is a single-center retrospective non-randomized study, focused only on severe COVID-19 pneumonia. Therefore, the impact of physiotherapy on asymptomatic infection or mild-moderate COVID-19 pneumonia was not evaluated. Second, during the first waves of the pandemic, the lack of knowledge regarding subject and healthcare worker safety, coupled with the lack of resources, conditioned a prioritization of physiotherapy in intensive care units. Consequently, the effect of a systematic and protocolized application of physiotherapy on all subjects cannot be extrapolated. Finally, differences between subjects that received physiotherapy earlier or later could be have been influenced by dissimilarities in the severity of the acute phase, such as the previous physical activity of the patient, which were not recorded (17). However, there are few published studies regarding early mobilization in critically ill subjects (47) and even fewer on the acute phase of severe COVID-19 pneumonia (51). Thus, this study has interesting results, such as safety for subjects and healthcare workers. Besides, early physiotherapy could shorten the time until sitting position and the length of hospital stay in subjects with more severe illness. In this regard, the results of this study results could be useful to develop prospective well-designed randomized studies in rehabilitation in the acute phase of COVID-19 pneumonia.

## Conclusion

There is no evidence that early or non-early physical therapy could be unsafe for healthcare workers or produce adverse effects in patients with severe COVID-19 pneumonia. Furthermore, physical therapy could reduce the time from admission until sitting position and length of hospital stay.

## Data Availability Statement

The raw data supporting the conclusions of this article will be made available by the authors, without undue reservation.

## Ethics Statement

The studies involving human participants were reviewed and approved by Hospital Ethics Committee (reference code PR168/20). Written informed consent for participation was not required for this study in accordance with the national legislation and the institutional requirements.

## Author Contributions

JB-M contributed to literature search, data collection, study design, data analysis, manuscript preparation, and manuscript review. AL-G contributed to data collection, manuscript preparation, and manuscript review. AA, FT, and LM contributed to data collection. VV-Z and SS contributed to study design and manuscript review. GB contributed to data collection and manuscript review. MM-M and GS-C contributed to study design, data analysis, manuscript preparation, and manuscript review. RP contributed to study design, manuscript preparation, and manuscript review. All authors contributed to the article and approved the submitted version.

## Conflict of Interest

The authors declare that the research was conducted in the absence of any commercial or financial relationships that could be construed as a potential conflict of interest.

## Publisher’s Note

All claims expressed in this article are solely those of the authors and do not necessarily represent those of their affiliated organizations, or those of the publisher, the editors and the reviewers. Any product that may be evaluated in this article, or claim that may be made by its manufacturer, is not guaranteed or endorsed by the publisher.
