# Supplementary material for: Effects of Early Physical Therapy and Follow-Up in Acute Severe Coronavirus Disease 2019 Pneumonia: A Retrospective Observational Study
Source: Front Med (Lausanne). 2022 Apr 11;9:866055. doi: 10.3389/fmed.2022.866055 (PMC9035928; doi:10.3389/fmed.2022.866055)
Supplement: Supplementary file 1 [file Data_Sheet_1.pdf]

Supplementary Material

Supplementary material 1 – REHABILITATION PROGRAM

The rehabilitation program included musculoskeletal and respiratory exercises. Two programs were designed: the first one (exercises 1-6 of appendix 1) was for subjects on-bed or sitting without standing capacity; the second program (exercises 1-3 and 7-10) was started once assisted standing was tolerated.

Pulmonary rehabilitation aimed to 1) improve the strength of the respiratory musculature; 2) increase pulmonary recruitment to improve pulmonary ventilation and oxygenation; 3) accelerate tolerance to physical activity and walking; 4) reduce basal atelectasis that hypothetically could favor fibrotic lung process; 5) improve drainage of secretions, mainly in tracheostomized subjects. Breathing exercises included guided breathing and spirometry with Threshold PEP and bottle PEP

Musculoskeletal rehabilitation aimed at 1) improving the strength of the lower limb musculature to initiate sitting, standing and early walking, then making it safe and autonomous; 2) improving the strength of the upper limb musculature to assist in transfers; 3) improving respiratory mechanics; 4) cardiovascular training. Musculoskeletal exercises included elevations of arms and legs without and with resistance, squats, buttock abduction and push-ups arms against a wall.

Rehabilitation was carried out daily in the ICU, sometimes morning and afternoon depending on the availability of physiotherapists, and from Monday to Friday in semi-critical care once a day until discharge from hospital.

COVID-19 respiratory physiotherapy is considered a high-risk technique because it generates aerosols and micro-droplets stables and contagious for 3 hours (1,2). Therefore, following the recommendations of the main medical societies (3–6), the physiotherapist carried out the rehabilitation with: filtering face pieces *(FFP)* 3 mask, a disposable long sleeve waterproof gown, double glove and eye protection.

Exercise 1. Guided Ventilation

a. Place your hands on each side of your lower chest (last ribs).

b. Slowly take in air through the nose.

c. Watching the hands move with the chest as air enters the lungs.

d. Hold your breath for 2 seconds.

e. Slowly let the air go through the mouth with pursed lips (as if to make the air "dance" from a candle without blowing it out) and observe how the hands move again with the chest as the air comes out of the lungs.

f. Rest for 5 seconds and do the exercise again.

Exercise 2. Incentive spirometry with Threshold PEP.

a. Breathe in slowly through your nose with your mouth closed.

b. Hold for 3-5 seconds at the end of this inspiration.

c. Take the Threshold PEP® device, place the skewer in your mouth, and slowly. breathe out through your mouth until your lungs are empty.

d. Do 5 repetitions in a row. Do not run, the maneuvers should be slow and paused.

e. Rest between 5 and 30 seconds between repetitions, depending on the tiredness.

Exercise 3. Incentive spirometry with a water bottle (PEP bottle)

a. Breathe in slowly through your nose with your mouth closed.

b. Hold for 3-5 seconds at the end of this inspiration.

c. Put the tube in the mouth.

d. Blow slowly through the tube, but with enough force to make air bubbles in the water in the bottle.

e. Hold these bubbles for about 5 seconds.

f. Rest between 5 and 30 seconds between repetitions, depending on tiredness.

Exercise 4. Elevation of arms (without weight)

a. Sitting well in the chair, with your hands on your knees.

b. Raise your arms while taking in air through your nose.

c. Then lower them while expelling the air through the mouth.

Exercise 5. Opening and closing the arms

a. Well seated in the chair; arms stretched down outside the armrests.

b. Raising our arms as if we want to touch our hands above our head while taking in air through our nose.

c. Lowing them back to the starting position while releasing air through the mouth.

Exercise 6. Raising the legs

a. Well seated in the armchair.

b. Lift one leg, leaving the knee straight, hold the contraction for 3 seconds.

c. Lower the leg to the starting position.

d. Alternate one leg with the other.

Exercise 7. Squats

a. Stand up, with your back against the wall.

b. Go down slightly bending your knees, dragging your back along the wall.

c. Once we do not find ourselves with our knees slightly bent, hold the position for three seconds.

d. Return to the starting position.

Exercise 8. Buttock abduction

a. Stand up, with our arms resting on the wall or over some surface to stabilize our posture.

b. Keeping our backs straight at all times.

c. Lift your leg sideways up to a maximum of 45º.

d. Hold the position for three seconds.

e. Return to the initial position.

Exercise 9. Push-ups arms against a wall

a. Stand up, with our arms stretched out and leaning against the wall at shoulder height.

b. Let the weight of our body fall slightly forward, taking in air through our nose (as if we wanted to touch the wall with our forehead).

c. Return to the starting position by releasing the air through the mouth while we make the effort.

Exercise 10. Elevation of arms (with weight. 1 kg approximately, according to individual tolerance).

a. Sitting well in the chair, with your hands on your knees.

b. Raise your arms with both weights in your hands while taking air through your nose.

c. Do not raise your arms beyond shoulder height.

d. Then lower them while expelling the air through the mouth.

Bibliography

1. Bahl P, Doolan C, de Silva C, Chughtai AA, Bourouiba L, MacIntyre CR. Airborne or Droplet Precautions for Health Workers Treating Coronavirus Disease 2019? J Infect Dis [Internet]. 2020 Apr 16 [cited 2021 Feb 18]; Available from: https://academic.oup.com/jid/advance-article/doi/10.1093/infdis/jiaa189/5820886

2. van Doremalen N, Bushmaker T, Morris DH, Holbrook MG, Gamble A, Williamson BN, et al. Aerosol and Surface Stability of SARS-CoV-2 as Compared with SARS-CoV-1. N Engl J Med [Internet]. 2020 Apr 16 [cited 2020 Jun 20];382(16):1564–7. Available from: http://www.nejm.org/doi/10.1056/NEJMc2004973

3. Gómez AA, López AM, Villelabeitia K, Morata AB. Actualización en rehabilitación respiratoria en el paciente con COVID-19 DE SORECAR. 2020;1–13.

4. Área de Fisioterapia Respiratoria de SEPAR. Fisioterapia respiratoria en el manejo del paciente con COVID-19: recomendaciones generales. Versión 2. 20 de abril 2020.

5. Lazzeri M, Lanza A, Bellini R, Bellofiore A, Cecchetto S, Colombo A, et al. Respiratory physiotherapy in patients with COVID-19 infection in acute setting: a Position Paper of the Italian Association of Respiratory Physiotherapists (ARIR). Monaldi Arch Chest Dis [Internet]. 2020 Mar 26 [cited 2020 Jun 13];90(1):163–8. Available from: https://www.monaldi-archives.org/index.php/macd/article/view/1285

6. Zhao H-M, Xie Y-X, Wang C. Recommendations for respiratory rehabilitation in adults with coronavirus disease 2019. Chin Med J (Engl) [Internet]. 2020 Jul 5;133(13):1595–602. Available from: https://journals.lww.com/10.1097/CM9.0000000000000848
